# Changes in body dissatisfaction and dieting, and its association with mental health problems among Japanese eighth year adolescents: a 11-year time trend study

**DOI:** 10.1186/s13034-025-00934-0

**Published:** 2025-06-18

**Authors:** Shoko Hamada, Hitoshi Kaneko, Masayoshi Ogura, Andre Sourander

**Affiliations:** 1 Department of Psychosociology, School of Arts and Letters, Meiji University, 1-1 Kanda-Surugadai Chiyoda-Ku, Tokyo, 101-8301 Japan; 2 Psychological Support and Research Center for Human Development, Nagoya University, Furo-Cho, Chikusa-Ku, Nagoya, 464-8601 Japan; 3 Naruto University of Education, 748 Nakashima, Takashima Naruto-Cho, Naruto-City, Tokushima 772-8502 Japan; 4 Department of Child Psychiatry, Turku University Hospital, University of Turku, 20014, 20520 Turku, Finland; 5 INVEST Research Flagship, University of Turku, Turku, Finland

**Keywords:** Dieting, Body image, Body dissatisfaction, Mental health, Adolescents

## Abstract

**Background:**

This study examined the time trends of body dissatisfaction and dieting among Japanese adolescents and the possible associated mental health problems.

**Methods:**

Two cross-sectional studies were conducted in 2011 and 2023 in the same 17 schools in one prefecture of Japan. Participants comprised 1865 (a 92.8% response rate) and 1174 (a 71.5% response rate) eighth grade students, who participated in the 2011 and 2023 surveys, respectively. Body dissatisfaction and dieting were assessed via a scale that assesses eating behavior and dieting. Mental health problems were assessed via the Strengths and Difficulties Questionnaire (SDQ).

**Results:**

The total score on items for eating behavior and dieting did not change between 2011 and 2023. Moreover, no significant interaction was observed between gender and years surveyed. The associated factors measured in the SDQ were similar in 2011 and 2023. An item-level analysis revealed an increase in score of items related to dieting, the fear of getting fat, and wanting to lose weight by not eating among male adolescents. Furthermore, among females, an increase was observed in items on experiences of losing weight and a decrease in items related to dissatisfaction with body image.

**Conclusions:**

The extent of body dissatisfaction and dieting remained stable among Japanese adolescents from 2011 to 2023. Those who were dieting or had eating problems reported higher emotional problems. More males reported dieting and fear of getting fat in the 2023 survey than was reported in 2011. Future studies should conduct surveys in Asian countries other than Japan, including low- and middle-income countries.

## Background

Body dissatisfaction is defined as a person’s negative thoughts and feelings about their body [1]; this state is most often observed from adolescence [2]. Eating disorders, such as anorexia nervosa, can begin during the early adolescent period [3]. Restrictive dieting is viewed as a behavioral indicator of body dissatisfaction [1]. It is also a risk factor of later disordered eating behavior [3]. Eating disorders, such as anorexia and bulimia nervosa, are associated with a higher mortality rate than other psychiatric problems [4]. To prevent issues related to body dissatisfaction and dieting from exceeding diagnostic levels, the trends of these disorders must be investigated among community-dwelling adolescents.

Time-trend studies enable us to determine how factors such as mental health problems among adolescents change over time. However, increases or decreases in these challenges vary, based on the country, time range, and behavior examined. A study in Finland, for example, reported a decrease of these behaviors among females over the period of 20 years [5], while another study reported an increase of compensatory behavior among females to reduce weight after over-eating [6]. A time-trend study in Sweden reported that dieting had become increasing prevalent among both male and female adolescents [7]. Another study in Iceland reported an increase in restrictive dieting among males [8]. Hence, the time shifts of these behaviors differ among studies. To the best of our knowledge, time-trend studies on body dissatisfaction and dieting behavior among adolescents have been mainly conducted in European countries, while studies from Asian countries are limited.

Furthermore, these problems have been observed more among females than males. While females consider thinness as more important, males think of muscularity as vital [1, 9]. Regarding body image among Japanese adolescents, female and male adolescents tend to overestimate and underestimate their weight, respectively [10]. Although the prevalence was lower than among female adolescents, male adolescents also reported problems of body dissatisfaction and dieting [11]. Some pile studies have examined these problems among males. For example, eating disorders have been observed among males during puberty [12], and after puberty, males with normal weight try to gain weight [13]. Another study reported that although the prevalence was lower than among females, the severity of weight control among males matched that of females [14]. Regarding Japanese male adolescents, Maezono et al. [15] reported that they had worse body image and higher eating distress compared with Finnish adolescents. Furthermore, factors associated with these problems, such as negative emotions, were similar in males to those of female adolescents [11].

Considering the dearth of related studies in Asian cultures, this research aimed to explore the time-trend shifts in body dissatisfaction and dieting among adolescents from 2011–2023 in the same school districts in Japan. Additionally, we compared the results with those of previous cross-sectional studies that used similar designs, methods, and assessment procedures. Primarily, we aimed to equate body dissatisfaction and dieting by comparing two cross-sectional studies focusing on gender differences, while also examining associated factors. We investigated whether body dissatisfaction, dieting, and their associated psychological problems changed over a decade. We also examined time-shifts of the prevalence of each behavior among females and males separately. We focused on how the prevalence of each item changed among male and female adolescents over the decade. Our study adds an important dimension to previous studies, in the form of novel data on Asian adolescents.

Our research questions were:

1. Has the extent of body dissatisfaction and dieting changed among females and males in 2023, compared with 2011?
2. Are factors associated with body dissatisfaction and dieting the same in 2023 as in 2011?
3. Has the prevalence of each item of body dissatisfaction and dieting changed in 2023, since 2011?

## Methods

### Participants and study design

The survey was conducted in Tokushima-Prefecture, Japan, and included 17 local junior high schools. Participating schools were chosen from a pool of 96 institutions, with assistance from the local education board. Data were collected in February and March 2011 and February and March 2023. Details of data obtained in 2011 are described in previous related works [15–18].

In both the 2011 and 2023 surveys, students voluntarily enrolled and did not receive any reward for participation. They were informed that the study was conducted to promote adolescent mental health, and were provided with the researchers’ email address if they required additional information regarding the study or any help with their mental health.

In the 2011 survey, surveys were distributed to 2010 participants, of which 1,865 (92.8% response rate) were returned. Of these, 25 (1.3%) did not specify their gender. Hence, data from 1840 students (946 females and 894 males) were analyzed. Participants’ average age was 13.9 years (SD 0.2; range 13–14).

In the 2023 survey, parents were informed of the survey. If they did not want their children to participate, they were asked to submit their child’s name. Thus, passive informed consent was obtained from the parents. In the questionnaire packet, participants were asked whether they agreed to participate. If they did not, they were asked not to complete the questionnaire and return them. At this stage, 35 students withdrew from the survey. Of the remaining questionnaires, 19 (1.6%) did not specify their gender. Hence, the data from 1155 students (533 females and 622 males) were analyzed. Participants’ average age was 13.9 years (SD 0.34, range 13–14).

**Fig. 1:**
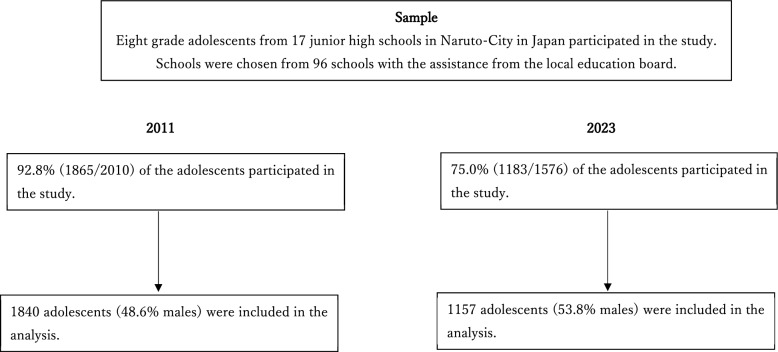
Flow chart

The attrition rate of the 2011 survey was 7.2%, while that of the 2023 survey was 30.5%. Figure 1 presents the flow chart for each survey.

Both population-based studies were approved by the Ethical Review Board of Nagoya University Graduate School of Education and Human Development (22–1816) and the leaders of the participating schools.

### Measures

#### Eating behavior

We used the scale developed by Koskelainen et al. [19] to assess body dissatisfaction and dieting behavior. Dieting, in this context, referred to unhealthy behavior to reduce weight such as excessive restriction on food intake. This scale has been shown to have sufficient validity, reliability, and internal consistency [5]. The internal consistency of this scale in the current study was satisfactory, with a Cronbach’s alpha of 0.83 in 2011 and 0.85 in 2023. Participants responded to the items on a 3-point Likert scale that included 0 = Not true, 1 = Somewhat true, and 2 = Certainly true. Item responses were summed to yield a total score for dieting and eating behaviors (range 0–18), with higher scores indicating more problems regarding eating behaviors. In the 2011 survey, two items; “I have deliberately vomited after having eaten” and “I have used pharmaceuticals to control my weight (appetite restricting drugs, laxatives, or diuretics)” were excluded owing to the ethical concerns voiced by the local education board. Thus, in this study, this scale was calculated with nine items.

#### Mental health

We used the Japanese version of Strength and Difficulties Questionnaire (SDQ) [20]. The SDQ is well-established in Japan [21], and comprises five subscales: emotional symptoms, conduct problems, hyperactivity/inattention, peer relationship problems, and prosocial behavior. Each subscale comprised five items. Participants rated the items on a 3-point Likert scale that included 0 = Not true, 1 = Somewhat true, and 2 = Certainly true. Scores for each subscale were calculated and higher scores indicated greater problems.

### Data analysis

To compare the total score of dieting and eating behaviors, an analysis of variance (ANOVA) was conducted with “gender” and “year surveyed” as the dependent variables.

To examine the association between gender and subscales of the SDQ on body dissatisfaction and dieting, separate multiple liner regression analyses were conducted for data from 2011 and 2023. “Gender” and the SDQ subscales were entered as independent variables and “total score of dieting and eating behavior” as the dependent variable.

To compare the frequency of body dissatisfaction and dieting in the 2011 and 2023 surveys, chi-squared tests were conducted for each gender. Responses of dieting and eating behavior were calculated as “yes” for answers of 2 (certainly true) and 1 (somewhat true), and as “no” for answers of 0 (not true). A chi-squared test was conducted for each item. Statistical analyses were conducted with using IBM SPSS 27.

## Results

### Time-shifts of body-dissatisfaction and eating problems

**Fig. 2:**
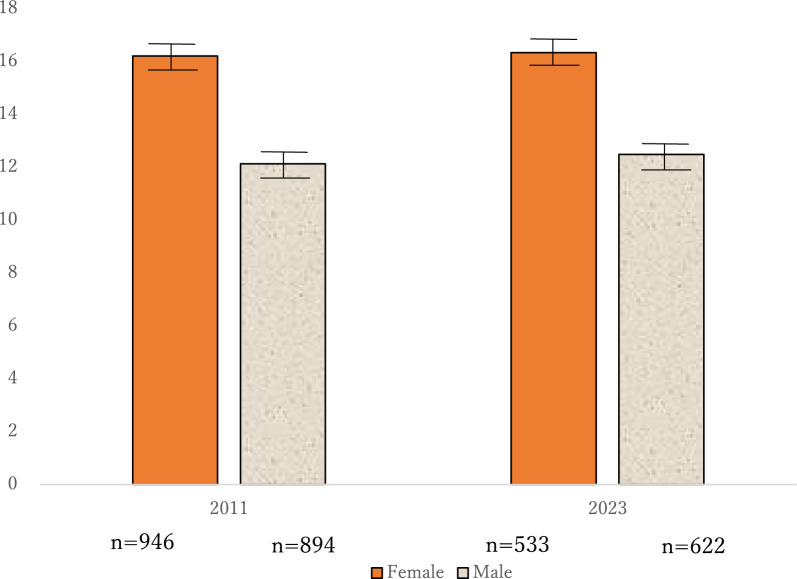
Total scores of self-reported body dissatisfaction and dieting in 2011 and 2023 Note. Error bars represent the standard error of the mean

Figure 2 presents the result of the ANOVA. The results of ANOVA revealed no significant interaction between gender and year surveyed (*F* (1, 2940) = 0.63, *n.s.*). Moreover, no significant main effect was observed in the year surveyed, which means that the scores of body dissatisfaction and dieting behavior had not changed between 2011 (Female M = 16.2, SD = 4.09, SE = 0.13; Male M = 12.1, SD = 3.17, SE = 0.11) and 2023 (Female M = 16.3, SD = 4.54, SE = 0.20; Male M = 12.48, SD = 3.27; SE = 0.13) (*F* (1, 2940) = 2.85, *n.s.*). Significant main effect was observed in terms of gender (*F* (1, 2940) = 772.13, *p* < 0.001), which indicates that females scored higher on these problems than males.

### Association of eating problems and dieting, and mental health

The means and standard deviations of the SDQ subscale among female and male participants in the 2011 and 2023 surveys are shown in Table 1.

**Table 1:** Means and standard deviations of SDQ subscales in the 2011 and 2023 surveys

	2011 survey				2023 survey			
	Female		Male		Female		Male	
	Mean	SD	Mean	SD	Mean	SD	Mean	SD
Emotional problems	4.35	2.64	2.88	2.28	4.56	2.57	3.04	2.36
Conduct problems	2.30	1.53	2.60	1.67	1.79	1.43	2.12	1.53
Hyperactivity/inattention	4.02	2.11	4.25	2.07	3.77	2.27	3.96	2.19
Peer relationship problems	2.32	1.63	2.62	1.75	2.21	1.64	2.23	1.57
Prosocial behavior	5.25	1.92	4.93	1.99	6.17	1.96	5.47	2.07

*SD* Standard deviation, *SDQ* strengths and difficulties questionnaire

Correlations were calculated separately for the 2011 and 2023 data. Table 2 presents the correlations among 2011 data, and Table 3 presents the correlations among 2023 data. Significant correlations were observed among dieting and eating behavior and gender (dummy coded; Female = 1, Male = 2) (*r* = − 0.49, *p* < 0.001), emotional problems (*r* = 0.46, *p* < 0.001), conduct problems (*r* = 0.18, *p* < 0.001), hyperactivity/inattention (*r* = 0.19, *p* < 0.001), and prosocial behavior (*r* = 0.13, *p* < 0.001). In the 2023 data, significant correlations were also observed among dieting and eating behavior and gender (dummy coded; Female = 1, Male = 2) (*r* = -0.40, *p* < 0.001), emotional problems (*r* = 0.40, *p* < 0.001), conduct problems (*r* = 0.18, *p* < 0.001), hyperactivity/inattention (*r* = 0.18, *p* < 0.001), peer relationship problems (*r* = 0.12, *p* < 0.001), and prosocial behavior (*r* = 0.19, *p* < 0.001).

**Table 2:** Correlation coefficients in each scale for 2011 data

		1		2	*p*-value	3	*p*-value	4	*p*-value	5	*p*-value	6	*p*-value	7	*p*-value
1	Gender (Female = 1, Male = 2)	–		– 0.49	0.001	– 0.28	0.000	0.09	0.000	0.06	0.000	0.08	0.000	– 0.09	0.000
2	Body dissatisfaction and dieting			–		0.46	0.000	0.18	0.000	0.19	0.000	0.04	*n.s*	0.13	0.000
3	Emotional symptoms					–		0.25	0.000	0.29	0.000	0.25	0.000	0.11	0.000
4	Conduct problems							–		0.38	0.000	0.29	0.000	0.11	0.000
5	Hyperactivity/inattention									–		0.18	0.000	– 0.22	0.000
6	Peer relationship problems											–		– 0.19	0.000
7	Prosocial behavior													–	

In the first row, 1 = Gender, 2 = Body dissatisfaction and dieting, 3 = Emotional symptoms, 4 = Conduct problems, 5 = Hyperactivity/inattention, 6 = Peer relationship problems, 7 = Prosocial behavior; *n.s.* = not significant

**Table 3:** Correlation coefficients in each scale for 2023 data

		1	*p*-value	2	*p*-value	3	*p*-value	4	*p*-value	5	*p*-value	6	*p*-value	7	*p*-value
1	Gender (Female = 1, Male = 2)	–		− 0.40	0.000	− 0.25	0.000	0.11	0.000	0.04	0.000	0.04	0.000	− 0.16	0.000
2	Body dissatisfaction and dieting			–		0.40	0.000	0.18	0.000	0.19	0.000	0.12	*n.s*	0.19	0.000
3	Emotional symptoms					–		0.22	0.000	0.35	0.000	0.34	0.000	0.01	0.01
4	Conduct problems							−		0.41	0.000	0.28	0.000	− 0.17	0.000
5	Hyperactivity/inattention									–		0.22	0.000	− 0.26	0.000
6	Peer relationship problems											–		− 0.13	0.000
7	Prosocial behavior													–	

In the first row, 1 = Gender, 2 = Body dissatisfaction and dieting, 3 = Emotional symptoms, 4 = Conduct problems, 5 = Hyperactivity/inattention, 6 = Peer relationship problems, 7 = Prosocial behavior; *n.s.* = not significant

Multiple regression analyses were performed separately for 2011 and 2023 data. Table 4 presents the results for 2011 data and Table 5 for 2023 data. In the 2011 and 2023 data, females scored higher for dieting and eating behaviors than males (β = − 0.41, *p* < 0.001 in 2011; β = − 0.34, *p* < 0.001 in 2023). Emotional problems was also significantly correlated with the body dissatisfaction and dieting behavior scores in both the surveys (β = 0.29, *p* < 0.001 in 2011; β = 0.23, *p* < 0.001 in 2023). β value of other variables, such as conduct problems (β = 0.13, *p* < 0.001 in 2011; β = 0.15, *p* < 0.001 in 2023), hyperactivity/inattention (β = 0.10, *p* < 0.001 in 2011; β = 0.11, *p* < 0.001 in 2023), peer relationship problems (β = − 0.04, *p* < 0.05 in 2011), and prosocial behavior (β = − 0.08, *p* < 0.01 in 2011; β = − 0.16, *p* < 0.001 in 2023), were low.

**Table 4:** Results of the multiple regression analysis with dieting and eating behavior as dependent variable for 2011 data (N = 1840)

	Adjusted β	*p*-value	Adjusted R^2^
**Gender (female = 1, male = 2)**	**− 0.41**	**0.000**	
**Emotional symptoms**	**0.29**	**0.000**	
Conduct problems	0.13	0.000	
Hyperactivity/inattention	0.10	0.000	
Peer relationship problems	**− **0.04	0.047	
Prosocial behavior	**− **0.08	0.001	0.39

Bold letters are the items that are factors that are strongly associated with dieting and eating behavior

**Table 5:** Results of the multiple regression analysis with dieting and eating behavior as dependent variable for 2023 data (N = 1155)

	Adjusted β	*p*-value	Adjusted R^2^
**Gender (female = 1, male = 2)**	**− 0.34**	**0.000**	
**Emotional symptoms**	**0.23**	**0.000**	
Conduct problems	0.15	0.000	
Hyperactivity/inattention	0.11	0.000	
Peer relationship problems	–	–	
Prosocial behavior	**− **0.16	0.000	0.29

Bold letters are the items that are factors that are strongly associated with dieting and eating behavior

### Time shifts of each of dieting and eating behavior among females and males

The time-shifts in the prevalence of dieting and eating behaviors were different for females and males. Among females, the prevalence of “I have lost weight by not eating in a short period of time” (*χ*^2^(1) = 7.48, *p* < 0.001) (13.0% in 2011; 18.3% in 2023) increased and “I am not happy with my body” (*χ*^2^(1) = 13.57, *p* < 0.001) (85.2% in 2011; 77.6% in 2023) decreased, from 2011 to 2023. Among males, an increase from 2011 to 2023 was observed on three items: “I have been on a diet” (*χ*^2^(1) = 19.52, *p* < 0.001) (10.5% in 2011; 18.5% in 2023), “I am afraid of getting fat” (*χ*^2^(1) = 21.83, *p* < 0.001), (29.3% in 2011; 41.3% in 2023), and “I have lost weight by not eating in a short period of time” (*χ*^2^(1) = 5.69, *p* < 0.05) (5.9% in 2011; 9.2% in 2023). Tables 6 and 7 present the results among the females and males, respectively.

**Table 6:** Results of the chi-squared test on dieting and eating behavior among female adolescents

	Not true				Somewhat true/True				*χ*^2^ test
Female	2011		2023		2011		2023		
	*n*	%	*N*	%	*N*	%	*n*	%	
Item 1: I would like to be thinner	125	13.3	88	16.6	813	86.7	443	83.4	*χ*^2^(1) = 2.88
									*n.s*
Item 2: I exercise a lot to avoid gaining weight	423	45.0	214	40.3	516	61.9	317	59.7	*χ*^2^(1) = 3.11
									*n.s*
Item 3: I have been on a diet	449	47.8	279	52.5	490	52.2	250	47.5	*χ*^2^(1) = 3.03
									*n.s*
Item 4: I am afraid of getting fat	307	32.8	159	30.0	630	67.2	371	70.0	*χ*^2^(1) = 1.19
									*n.s*
**Item 5: I have lost weight by not eating in a short period**	**816**	**87.0**	**433**	**81.7**	**122**	**13.0**	**97**	**18.3**	***χ***^**2**^**(1) = 7.48** ***p* < 0.001**
**Item 6: I am not happy with my body**	**139**	**14.8**	**119**	**22.4**	**800**	**85.2**	**412**	**77.6**	***χ***^**2**^**(1) = 13.57**
									***p* < 0.001**
Item 7: It terrifies me if I gain even a little weight	579	61.7	305	57.4	359	38.3	226	42.6	*χ*^2^(1) = 2.60
									*n.s*
Item 8: I am not always able to control my eating	467	49.8	242	45.6	470	50.2	289	54.4	*χ*^2^(1) = 2.47
									*n.s*
Item 9: I devour large amounts of food at one time	454	48.3	254	47.8	486	51.7	277	52.2	*χ*^2^(1) = 0.03
									*n.s*

Bold letters are the items for which a significant increase or decrease were found between 2011 and 2023

**Table 7:** Results of the chi-square test on dieting and eating behavior among male adolescents

Male	Not true				Somewhat true/True				*χ*2 test
	2011		2023		2011		2023		
	*n*	%	*n*	%	*n*	%	*n*	%	
Item 1: I would like to be thinner	601	68.6	397	63.8	275	31.4	225	36.2	*χ*2(1) = 3.74
									n.s
Item 2: I exercise a lot to avoid gaining weight	559	63.8	374	60.2	317	36.2	247	39.8	*χ*2(1) = 2.00
									n.s
**Item 3: I have been on a diet**	**783**	**89.5**	**506**	**81.5**	**92**	**10.5**	**115**	**18.5**	***χ*2(1) = 19.52**
									**p < 0.001**
**Item 4: I am afraid of getting fat**	**619**	**70.7**	**366**	**59.0**	**257**	**29.3**	**254**	**41.0**	***χ*2(1) = 21.83**
									**p < 0.001**
**Item 5: I have lost weight by not eating in a short period**	**825**	**94.1**	**564**	**90.8**	**52**	**5.9**	**57**	**9.2**	***χ*2(1) = 5.69**
									**p < 0.05**
Item 6: I am not happy with my body	459	52.3	327	52.6	418	47.7	295	47.4	*χ*2(1) = 0.01
									n.s
Item 7: It terrifies me if I gain even a little weight	769	87.8	527	84.9	107	12.2	94	15.1	*χ*2(1) = 2.67
									n.s
Item 8: I am not always able to control my eating	596	68.0	418	67.5	280	32.0	201	32.5	*χ*2(1) = 0.04
									n.s
Item 9: I devour large amounts of food at one time	554	63.2	418	67.2	322	36.8	204	32.8	*χ*2(1) = 2.50
									n.s

Bold letters are the items for which a significant increase or decrease were found between 2011 and 2023

## Discussion

We used data from two cross-sectional school-based surveys among Japanese adolescents and investigated the shifts in dieting, eating problems, and associated factors among adolescents from 2011 to 2023. The results of ANOVA indicated no interaction or main effect between the score of 2011 and 2023, which means that overall, the extent of dieting and eating problems were stable between 2011 and 2023, among both females and males. Furthermore, emotional problems were strongly associated with the subscales of the SDQ in both the 2011 and 2023 data. Moreover, more male adolescents reported “experience of being on a diet,” “fear of getting fat,” and “experience of losing weight by not eating” in 2023 than in 2011. Among females, the prevalence of “losing weight without eating” increased and “dissatisfaction with the body” decreased.

Comparison of the two cross-sectional studies in 2011 and 2023 revealed no significant changes in the total scores of dieting and eating problems among both females and males. Hence, the extent of these problems remained stable from 2011 to 2023 among Japanese adolescents. Body image and dieting behavior are often affected by cultural body ideals [1]. In many cultures, being slender is idealized among females and for males, being muscled is idealized [1]. The findings of this study—that the total scores of dieting and eating were stable between 2011 and 2023—might indicate that these ideals have not changed in over a decade in Japan. Similar results were observed among adolescents in Finland; in the study, the same body dissatisfaction and dieting scale as this study was utilized [5]. Moreover, a study in Cyprus [22] found no significant differences in their time-trend study over a decade. In line with the findings of other studies [5, 22], our results revealed that consistent dieting and eating problems were observed more among female than male Japanese adolescents, over the decade.

The results of the multiple regression analyses revealed that females were more likely to have dieting and eating problems both in 2011 and 2023. Emotional problems were also associated with dieting and eating problems. Although the *p*-values of conduct problems (2011 and 2023 data), hyperactivity/inattention (2011 and 2023 data), peer relationship problems (2011 data) and prosocial behavior (2011 and 2023 data) were significant, the adjusted βs were low. This indicates that emotional problems were strongly associated with the dieting and eating problems in both the 2011 and 2023 datasets, when controlling for gender. A previous study revealed that emotional instability mediates the association between social comparison and excessive eating [23]. Considering the previous study and the current study, social comparison with peers may be one factor affecting the association between emotional problems and eating problems. Future studies should include these variables to understand the mechanisms of the association of emotional problems and eating problems more accurately. The finding that females have more of these problems than males is consistent with those of studies conducted in Western countries. Neumark-Sztainer and Hannan [24] indicated that these problems were observed more among females than males, and were associated with emotional problems such as depression and stress. Moreover, negative emotions were associated with a desire for thinness in longitudinal study among Australian female adolescents [25]. Our findings add an important aspect to the research area that these findings are also applicable to Japanese adolescents [26].

Notably, an increase in dieting and body image was observed among males when each item was examined over 11 years. More male adolescents reported experiences of dieting, were afraid of getting fat, and reported experiences of losing weight by not eating in 2023 than in 2011. The desire to be thinner and dissatisfaction with their body image became more prevalent among male adolescents over this 11-year time period. A large time-trend study on adolescents’ body mass index (BMI) revealed that Japanese adolescents’ BMI has become healthier during the past 30 years [27]. However, our findings indicate that male adolescents had worse body image, and that more male adolescents had experienced dieting during this decade. An increase in dieting among males was also reported in Iceland [6]. Studies show that it is believed among male adolescents that being fat is considered to be weakness and lack of control [27]. Our results seem to indicate that more young adolescents have come to think this way during this decade in Japan; thus, they tend to avoid possibly gaining weight. Therefore, more attention should be paid to dieting and eating problems among males, as well as females. Our findings provide the valuable insight that dieting has become more prevalent among Japanese male adolescents over the last decade.

Regarding time-trend shifts in females, the number of adolescents who experienced weight loss with a short period of time without eating increased, while the number of adolescents dissatisfied with their body decreased. Many societal changes came about during this decade. For example, adolescents can obtain more information on dieting through online media than a decade ago. Moreover, they also share their own pictures on social media. Adolescents are therefore more sensitive about their appearance than they were a decade ago. Additionally, this survey was conducted after the COVID-19 pandemic. During the pandemic, adolescents’ activities were restricted, which may have led them to engage in excessive dieting, rather than exercising. Although many other changes came about during this decade, these societal changes might affect the increase in unfavorable dieting behaviors among adolescents. Regarding the decrease in dissatisfaction with their body among female adolescents, the reason remains unclear in this study. Future studies should investigate this perspective, and include the height and weight, to examine discrepancies with their actual size and body image. Regarding with dissatisfaction with their body, the same stable trend was observed among Finnish female adolescents over a period of 20 years, who lost weight over a short period of time [5]. Dieting and eating problems were observed more among females than males. Further research on body image and dieting among females is required.

### Strengths and limitations

Some strengths and limitations should be mentioned when interpreting our study. An important strength is that we conducted two surveys, 11 years apart, in the same school districts. This enabled us to compare the results in a linear and direct fashion. Second, we conducted a survey with numerous adolescents. To the best of our knowledge, this study was the first to examine body dissatisfaction and dieting among Japanese adolescents via a time-trend study. Third, we used reliable scales to assess the problems of eating and dieting and emotional and conduct problems. This enabled us to compare our findings with those of previous studies.

However, certain limitations must also be acknowledged. First, the survey was conducted in only one location in Japan. Moreover, the participants included only eighth grade students. To determine whether these results are generalizable to all Japanese adolescents, further surveys should be conducted in more areas and include adolescents of different ages. Second, we did not obtain information on height or weight to calculate BMI. Thus, we were unable to examine actual body shape and their body image. Future studies should include questions regarding BMI. Third, the scale we used for assessing body dissatisfaction and dieting behavior is not validated in Japan. Thus, the results should be interpreted with caution, in terms of whether data are representative of Japanese adolescents. Forth, the attrition rate was higher in the 2023 survey than in the 2011 survey. This may be due to procedural differences in obtaining participants’ consent; passive consent was obtained from parents in the 2023 survey, whereas this was not the case in 2011. Moreover, we did not have access to the demographic data for those who did not participate in the survey to assess non-response bias. Finally, we did not consider socioeconomic status and school environment, such as academic pressures or school atmosphere. Future studies should include these variables to capture the whole picture of dieting and eating behaviors among adolescents.

## Conclusion

Our study found that eating behavior and dieting problems were stable during a period of 11 years among Japanese adolescents. Body dissatisfaction and dieting were observed more among female adolescents than male adolescents. We found that these behaviors were associated with emotional problems, measured with the SDQ. Separate comparisons of the time shifts of males and females on each behavior revealed that male adolescents reported more eating behavior and dieting problems in 2023, than those in 2011. Future studies need to include more comprehensive variables regarding with socioeconomic status and factors associated with schools.

## Data Availability

The data that support the findings of this study are available from the corresponding author upon reasonable request.

## Abbreviations

ANOVA: Analysis of variance
BMI: Body Mass Index
COVID-19: Coronavirus disease 2019
SD: Standard deviation
SDQ: Strengths and difficulties questionnaire
